# Distinguishing Lipid Subtypes by Amplifying Contrast from J-Coupling

**DOI:** 10.1038/s41598-019-39780-4

**Published:** 2019-03-05

**Authors:** Ifeanyi K. Uche, Gigi Galiana

**Affiliations:** 1 Louisiana State University School of Veterinary of Medicine, Baton Rouge, LA USA; 2 Yale University, Radiology and Biomedical Imaging, New Haven, CT USA

## Abstract

Previous work has highlighted the complicated and distinctive dynamics that set signal evolution during a train of spin echoes, especially with nonuniform echo spacing applied to complex molecules like fats. The work presented here regards those signal patterns as codes that can be used as a contrast mechanism, capable of distinguishing mixtures of molecules with an imaging sequence, sidestepping many challenges of spectroscopy. For particular arrays of echo spacings, non-monotonic and distinctive signal evolution can be enhanced to improve contrast between target species. This work presents simulations that show how contrast between two molecules: (a) depends on the specific sequence of echo spacing, (b) is directly linked to the presence of J-coupling, and (c) can be relatively insensitive to variations in B0, T2 and B1. Imaging studies with oils demonstrate this phenomenon experimentally and also show that spin echo codes can be used for quantification. Finally, preliminary experiments apply the method to human liver *in vivo*, verifying that the presence of fat can lead to nonmonotonic codes like those seen *in vitro*. In summary, nonuniformly spaced echo trains introduce a new approach to molecular imaging of J-coupled species, such as lipids, which may have implications diagnosing metabolic diseases.

## Introduction

The behavior of coupled spins in a spin echo train have been known since the early days of MR (magnetic resonance) to depend on both spectroscopic parameters specific to the molecule and also on experimental parameters specific to the sequence^1–4^. Specifically, neglecting T2 decay (spin-spin relaxation), signal from a standard Carr-Purcell-Meiboom-Gill (CPMG) sequence yields signal at the nth echo peak, S(nτ), equal to:

$$S(n\tau )\propto \sum _{m=0}^{{2}^{N}({2}^{N}-1)}{c}_{m}\,\cos ({\omega }_{m}n)+c$$

where m indexes over each spin pair in the N-spin coupling network, and c_m_ and ω_m_ are constants, which depend on the J and Δω of each coupled spin pair as well as the echo spacing of the pulse sequence, τ. While this equation describes a simple sum of sinusoids, the complication is that c_m_ and ω_m_ are not straightforward functions of J, Δω and τ. In general, calculating these parameters requires knowledge of off-diagonal terms in the density matrix using average Hamiltonian theory or a numerical simulation. Analytic equations have only been published for small spin systems and a few extreme limits in echo spacing^1–4^.

Two approximations applicable at the extreme limits are widely known. When τ ≫ 1/J, ω_m_ = J of the spin pair. At the other limit, when τ ≪ 1/J, ω_m_ approaches zero, so all J-modulation disappears. The importance of this latter case resurfaced in MRI in the early 90 s, when it was noted that fat tissues appeared hyperintense on fast spin echo images, despite being relatively dark on conventional T2 weighted images^5–7^. Debate ensued over the origin of this phenomenon, but it is now generally accepted that the primary cause is suppression of J-modulation, which otherwise reduces spin echo intensity in complex molecules like lipids.

Typical fast spin echo (FSE) experiments applied to fats are not easily described by any simple formula; they involve echo spacings that do not fall at either simplifying extreme, as well as relatively complicated molecules. Stables *et al*. performed extensive simulations studying more complicated spin systems (A_3_B_2_ and A_3_B_2_C_2_) in echo train experiments of different echo spacing^3^. Those studies documented a wealth of erratic behavior, including a narrow range of τ where dynamics transitioned from relatively smooth evolution, where S(nτ) contains just a few frequency components, to “erratic” where a complex and variable number of frequency components contributed to the signal evolution. Furthermore, modeling the initial signal decay as a monoexponential T2, the authors found that “apparent T2” could vary by *two orders of magnitude* for τ between 10 and 40 ms. A study of effective T2 as a function of coupling strengths suggested that dependence on spectral parameters is similarly dramatic. Notably, this profound variability was observed even for these studies of uniformly spaced CPMG pulse sequences.

Previously reported studies have demonstrated distinctive dynamics that shape the signal evolution observed during a nonuniformly spaced train of spin echoes^8,9^. Several studies in the Warren group, extending quantum computing work by Uhrig^10,11^, highlighted the changes in dynamics that occur in going from a uniformly spaced sequence, like the classic CPMG, to a sequence with nonuniform spacing between echoes. From a T2 perspective these two sequences should yield identical echo magnitudes at the last echo. But theoretical work from quantum computing suggests they may differently refocus particular frequency components in the relaxation that lead to decoherence. However, *in vivo* experiments found the most dramatic differences between these two sequences in fat tissues, due to modulation of the J-coupling by the different spin echo trains^8^.

This previous work on irregularly spaced refocusing pulses focused on the magnitude of the last echo following a spin echo train preparation, but we hypothesized that the full evolution across the train of echoes may deserve further analysis. Since modulation across the echo train depends on spectroscopic parameters, signal evolution across a spin echo train may constitute a type of molecular fingerprint^12–14^. The work presented here tests whether this evolution is sufficient to identify and quantify different molecules. Unlike traditional MR spectroscopy methods to identify chemical components, static magnetic field (B0) inhomogeneity is unimportant in a spin echo train, which may have advantages for distinguishing species *in vivo*, especially those with similar chemical shift^15–17^. Furthermore, by tailoring the echo spacing, it may be possible to maximize contrast between species of interest, thereby providing a new contrast mechanism for molecular imaging *in vivo*.

The following studies present preliminary simulations and experiments demonstrating the idiosyncratic nature of behavior under irregularly spaced spin echo trains. Simulations show the power of echo spacing to tune contrast between two species, as well as effects from imperfect B0 (main magnetic field), T2, or B1 (radio frequency magnetic field). The oils tested experimentally with this method, diacetin and triacetin, provide simplified models of the glycerol backbones of diglycerides (DAG) and triglycerides (TAG), which have high clinical significance for the development of metabolic and liver disease^18–22^. We present results demonstrating experimental reproducibility of these signals, as well as the potential for quantitative analysis of the species present in each voxel. Finally, we present an initial demonstration in two human livers, one lean and one steatotic. In human studies, the echo train signal is nonmonotonic only in the steatotic liver, suggesting that the unusual signal evolution is driven by the coupled spin networks present in lipids.

## Results

### Simulations Results

**Figure 1:**
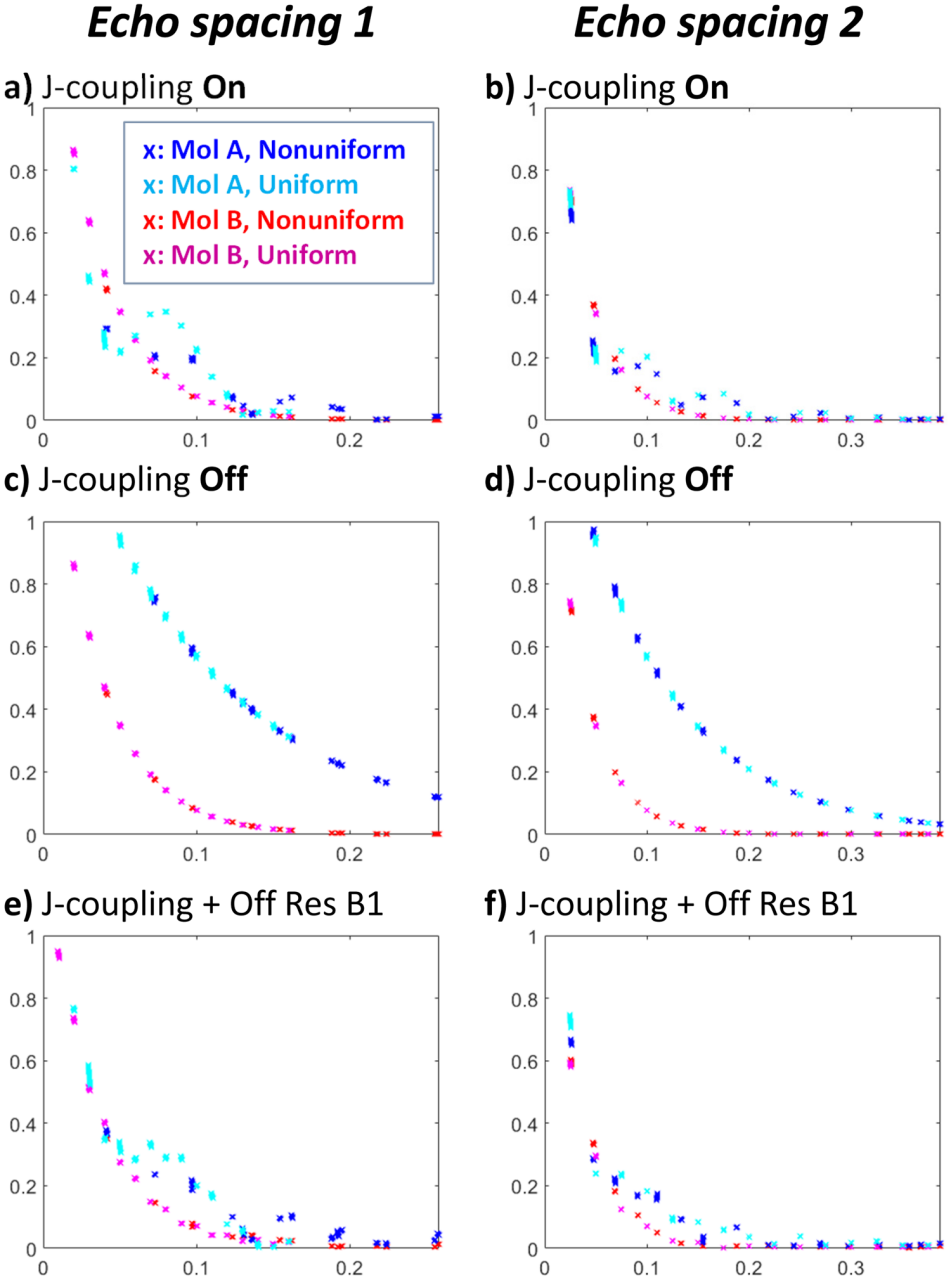
Simulations for two molecules with J-coupling present and removed. Panel (a) illustrates that echo spacing not only changes the sampling interval for an underlying evolution, it actually manipulates spin dynamics. This is most clearly seen by comparing the evolution of Mol A under nonuniform vs. uniform echo spacing (blue vs. cyan). Furthermore, different patterns of nonuniform echo spacing can greatly affect the contrast. Correlation between Mol A and B in a nonuniformly spaced echo train is 0.84 for the spacing shown in panel (a), but it is 0.96 for the spacing shown in panel (b). For uniformly spaced echo trains, the correlation is 0.95 in both panels. Panels (c,d) show that nonmonotonic dynamics disappear when we remove J-coupling between the spins. Finally, panels (e,f) show the potential of off-resonance effects, considering a flip angle of 170° for H3 of Mol B, and 180° for all others. This does affect the signal pattern, though the overall correlation remains similar (0.86 and 0.97, respectively).

Figure 1 shows an overlay of simulated echo train signals from two molecules. In the top panel, there is J-coupling amongst the spin networks in each molecule. The blue and cyan signals correspond to signal from Molecule A (Mol A) under a nonuniformly spaced and evenly spaced spin echo train, respectively. Similarly, the red and magenta signals correspond to Molecule B (Mol B) under a nonuniformly spaced and evenly spaced spin echo train, respectively. Showing each signal as a function of echo time verifies the basic principle that echo spacing does not simply change the sampling of an underlying evolution curve, it actively modifies the signal evolution. This is most easily seen in the gaps between blue and cyan signals in panel (a), which represent signal from the same molecule evolving under different spin echo trains. In the absence of J-coupling, as shown in panel (c), this is not observed; echoes sample the same underlying relaxation curve, regardless of spacing. Since these simulations do not take into account the full relaxation spectrum, the phenomenon described by Uhrig is not observed^8–11^.

Furthermore, in addition to changing the signals from an individual molecule, the contrast between two molecules can be modified by the spacing of an echo train, as seen by comparing panels (a,b). The correlation between signal from Mol A vs Mol B in these two different (nonuniform) echo spacings, is, respectively, 0.84 vs 0.96, suggesting these very similar experiments provide very different contrast. Both uniform spacings give a correlation of 0.95 between molecules, which provide little basis for distinguishing them. Finally, we consider off-resonance and the potential for imperfect refocusing of spins in panels (e,f), which show changes in the signal pattern, but little change in the correlation and contrast (0.86 and 0.97, respectively). However, this additional degree of freedom to manipulate signal pattern across echoes suggests this is another potential source of contrast, though it is not fully explored in the presented work.

**Figure 2:**
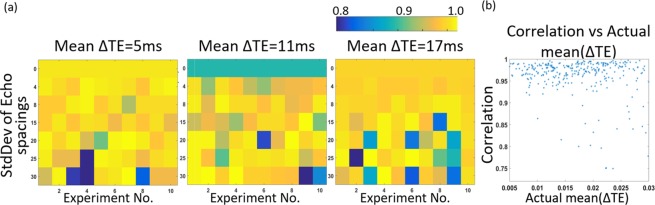
Contrast for different echo spacings. High contrast between two molecules depends on echo spacing but is not a simple function of mean echo spacing. In each row, a series of experiments were generated by choosing 16 echo spacings from a single distribution (mean and SD as labeled). The color in each block reflects correlation between signals, so low correlation can be interpreted as potential for high contrast between the molecules. Contrast is highly variable even for sequences with spacing taken from the same distribution. Panel (b) shows that contrast is also not correlated to the actual μ(ESP) of each experiment.

With a conventional MRI view of spin echo trains, one might expect contrast depends primarily on TE_max_ with some marginal dependence on mean echo spacing (ESP). This hypothesis is tested in Fig. 2, for 100 s of echo-train spacings. Each colored block of Fig. 2 represents a different pulse sequence based on a different 16 element array of echo spacings. Each row depicts ten candidate pulse sequences, each with echo spacings chosen randomly from the same distribution (labeled by the title and y-axis), thus expected to have comparable ESP. Note that experiments in the top row of each panel come from a distribution with standard deviation 0, corresponding to uniform spacing with ESP = μ(ESP). The color in each block reports the correlation between signals from Mol A and Mol B as acquired from a single experiment. This is equivalent to the correlation between the red and blue signals in Fig. 1 but for experiments with different echo spacing, and a low correlation implies good potential contrast. For nonuniformly spaced spin echo experiments, there is marked variability among sequences, with a range from 0.99 to 0.75, even between sequences with spacings taken from the same distribution. This suggests that contrasts between signals is sensitive to specific spacing patterns, not just the mean echo spacing.

Since ESP arrays taken from the same distribution may still differ significantly in their actual μ(ESP), we also plot signal correlation vs actual μ(ESP), not just μ(ESP) of the source distribution, for each sequence. Notably, this is equivalent to a plot of correlation vs actual maximum echo time, so this plot captures both the factors typically associated with contrast in a fast spin echo experiment. As in the previous panels, there is no simple trend between μ(ESP) and contrast. These results are exciting in that they show echo spacing is a powerful dimension for tuning J-coupling contrast, but they highlight the need to test many candidate spacings. These simulations suggests that contrast in nonuniformly spaced spin echo trains, measured as correlation between signals, is not a simple function of mean echo spacing or maximum echo time.

**Figure 3:**
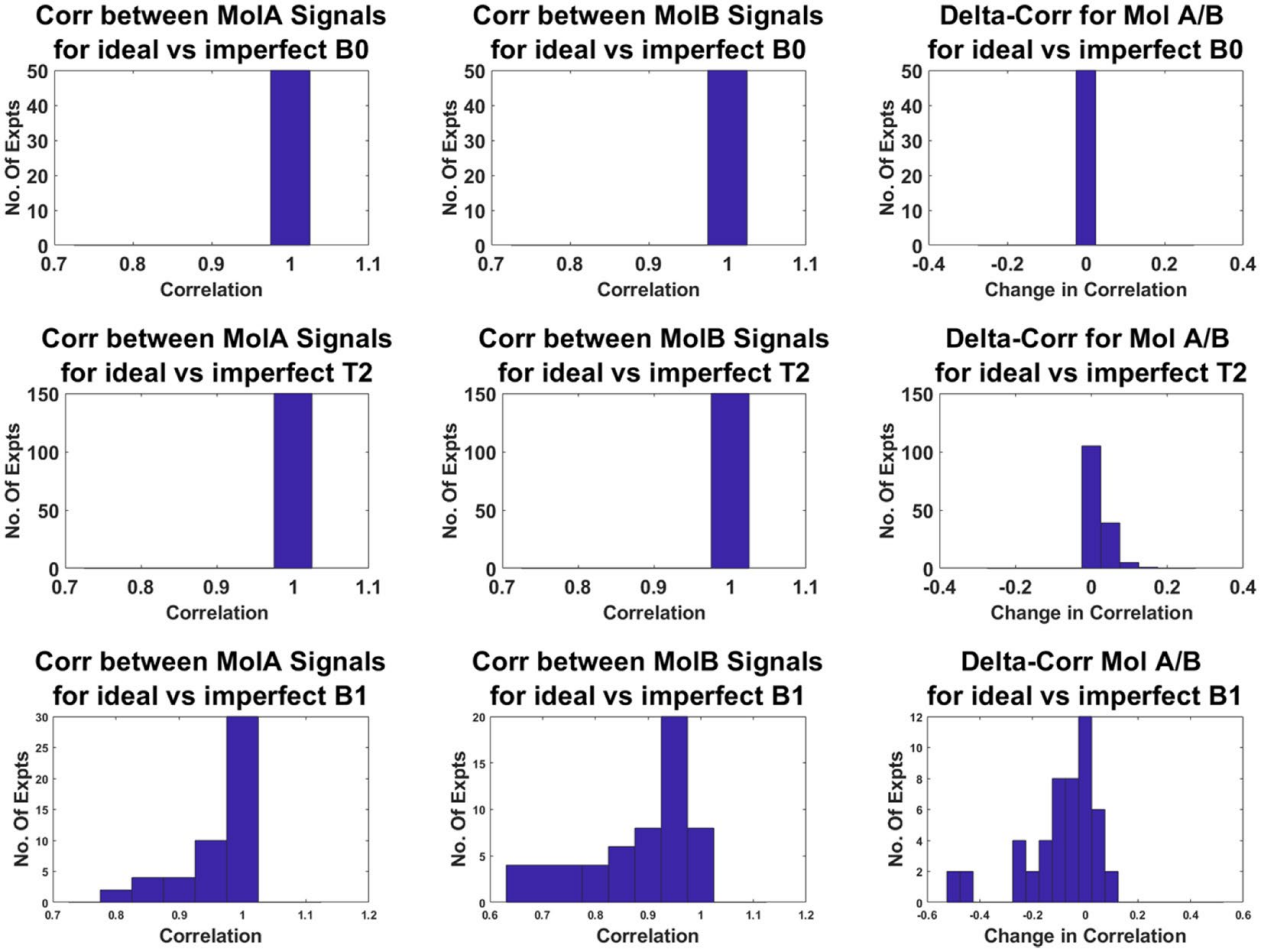
Correlation plots between simulated signal and imperfect B0, B1 and T2. For each echo spacing, the signal was simulated for ideal conditions and a range of nonideal conditions, recording the correlation of the ideal and nonideal signal, as well as any change in the contrast between signals from Mol A and Mol B. The correlations between nonideal and nonideal signals, for each molecule, are tabulated in histograms in the first two columns, while changes in the contrast between Mol A and Mol B are tabulated in histograms in the third column. Subsequent rows analyze nonidealities in B0, T2, and B1. This data shows that signals and contrast are entirely independent of B0 variability, and T2 variability only affects contrast for a minority of the sequences studied. B1 effects can be more significant, but many sequences are relatively insensitive.

Finally, simulations were performed to investigate the effects of B0, T2 and B1 variability. For each contrast generating pulse sequence from Fig. 2 (correlation < 0.9), signals were generated for both ideal and a series of non-ideal conditions, and Fig. 3 summarizes the observed changes to each signal and contrast. The first two columns show how a nonideality changes the signal from each molecule, while the third column shows how the nonideality changes the contrast between the molecules. Subsequent rows simulate variability in B0, T2, and B1.

As expected from theoretical considerations, signal evolution and contrast are wholly unaffected by global B0 shifts, an appropriate model for B0 in a refocused spin echo image. Since apparent T2 is easily dominated by J-coupling dynamics, changes in the underlying T2 also have minimal effect. Errors in B1 can be more significant, but for most sequences, the signal from each species changes very little. Furthermore, by using crusher gradients that select for a pure |Δp| = 2 coherence transfer, and by limiting analysis to region of interests (ROIs) with uniform B1, the influence of this confound can be reduced.

### Experimental Results

**Figure 4:**
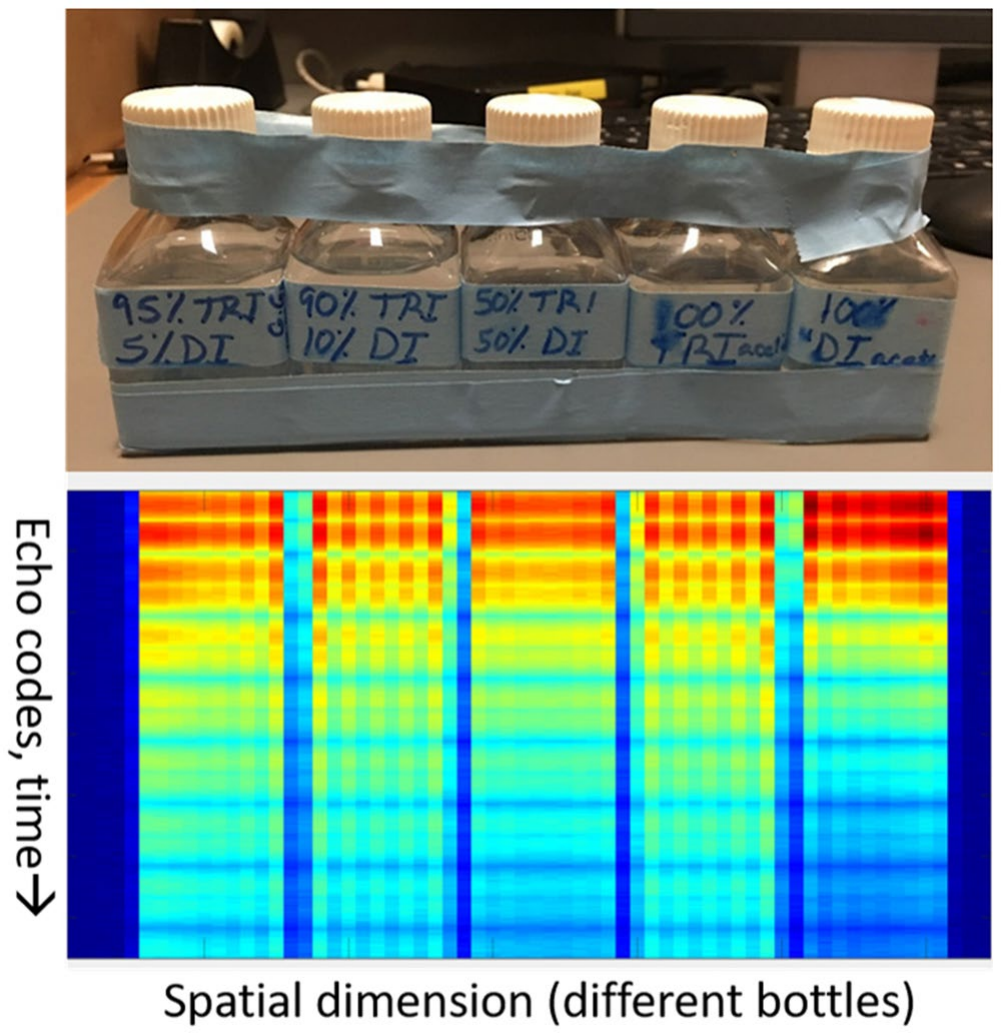
Experimental layout and spatial dimension of the different bottles. Bottles with varying concentrations of diacetin/triacetin were arranged as shown, and 1D spatial encoding was applied to generate projection images that isolated each bottle. After Fourier transform the data shows the 16 echoes from each spatial location, which was then averaged within each bottle to generate the multiecho code from each mixture.

**Figure 5:**
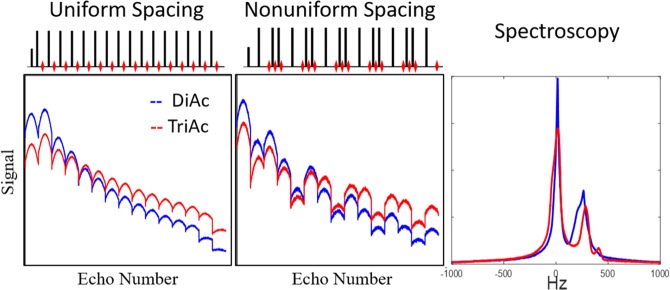
Comparison of signals from nonuniform echo trains, uniform echo trains, and spectroscopy. This verifies that nonuniform echo spacing can generate highly non-intuitive and non-monotonic evolution across a spin echo train, even in oils with very similar NMR spectra.

**Figure 6:**
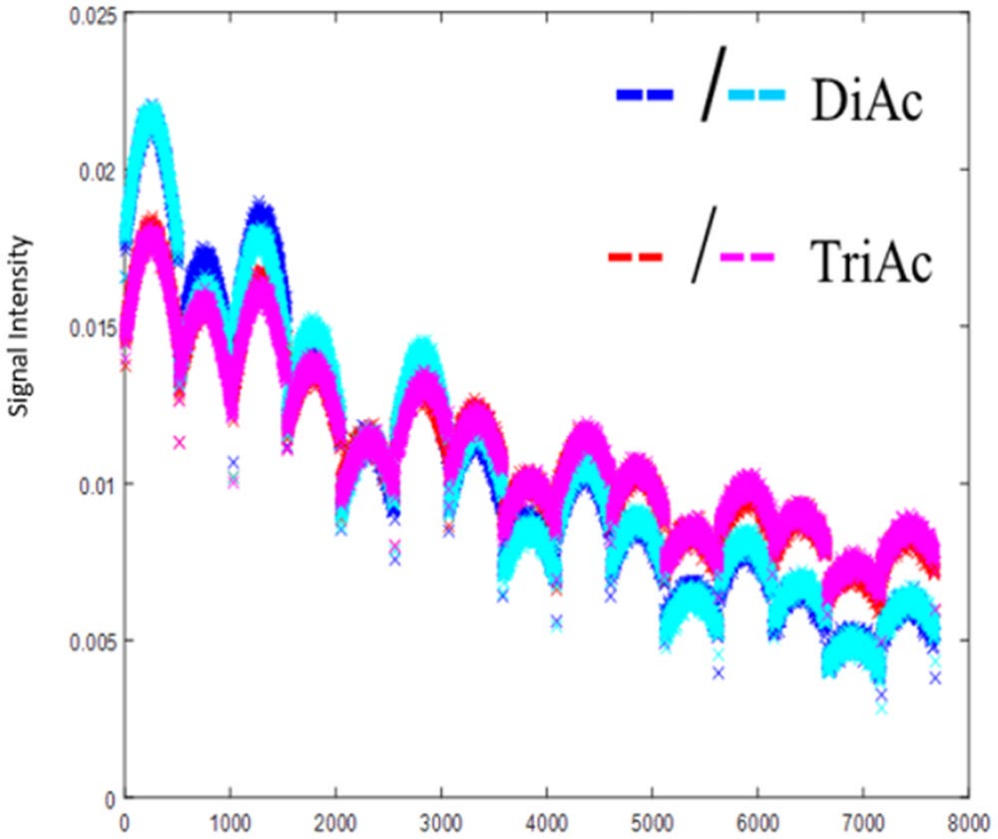
Signal reproducibility on different days. This figure shows the signals generated from each oil on different days and with bottles in different orientations. The experimental reproducibility supports simulations which suggest that echo train signals are not extremely sensitive to variations in B0 or B1.

The first experimental validations of this approach were tested on a series of oil mixtures made of diacetin and triacetin (100/0, 50/50, 10/90, 5/95 and 0/100), which respectively contain the same glycerol backbone structures as diglycerides and triglycerides. Since commercial diacetin is a mixture of diacetin, triacetin, and monoacetin, simulation results could not be directly translated and echo spacings were tested empirically. Figure 4 shows a typical experimental layout, as well as the signal following 1D spatial transform. After Fourier transform along the spatially encoded dimension, each column shows the 16 echoes associated with that location, with the five bottles arranged as shown above. Figure 5 shows the average signals from each pure oil as obtained by uniform spacing, nonuniform spacing (a pattern of 25-6-6 ESP), and single voxel spectroscopy. This experiment validates that irregularly spaced echo trains can generate highly nonintuitive and nonmonotonic signals experimentally. Figure 6 shows the results of the irregularly spaced echo train taken on different days with the sample in a different orientation, demonstrating the reproducibility of nonuniformly spaced echo patterns.

**Figure 7:**
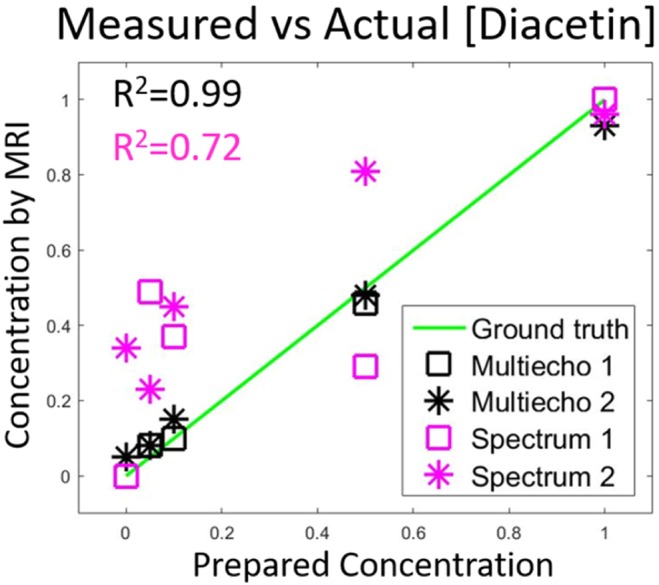
Signal quantification of multiecho codes of nonuniform spacing. Signals from each mixture were decomposed using the signals of pure oils as basis functions. The agreement with ground truth is excellent, even when using basis functions acquired on a different day and with a different sample orientation. A similar analysis based on spectroscopy (magenta data) does not perform as well.

Signal vectors from pure liquids, whether arising from spin echo trains or spectroscopy, can be regarded as basis functions that can be used to decompose the bulk signal into its constituents. This approach, using the pure diacetin/triacetin signals shown in Fig. 6, was used to quantify the composition of each vial for each experiment. Those results are shown in Fig. 7, which summarizes the concentrations estimated by either multiecho codes (black) or spectroscopy (magenta). Notably, the repeated data uses, as basis functions, the pure diacetin/triacetin signal acquired in the first experiment, which suggests the quantification is insensitive to typical variables in experimental setup (B0, B1, etc). The spin echo code yields a highly linear agreement with ground truth concentration, while the spectrum appears difficult to decompose in the presence of inhomogeneous broadening over the relatively large voxels. Furthermore, the reproducibility of these results over different days and orientations supports the simulation findings that spin echo codes are not overly sensitive to experimental imperfections, like errors in B0 or B1.

**Figure 8:**
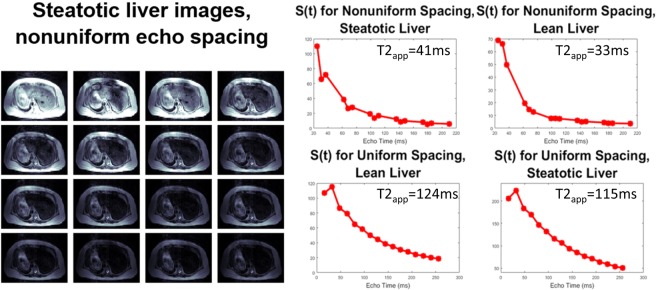
Feasibility in human liver. Images of mildly steatotic liver imaged with nonuniformly spaced echoes show feasibility of the sequence. Signal intensities for a liver ROI show nonmonotonic evolution, while a lean liver shows only decay.

Finally, the same nonuniformly spaced echo train which distinguished diacetin and triacetin was initially tested in two subjects with prospective acquisition correction (PACE) navigator self-gating. These studies aimed only to establish feasibility of the sequence, testing both a nonuniformly spaced 16 echo sequence (a 25-6-6 pattern found useful in diacetin/triacetin studies) and a comparable CPMG (ESP = 16 ms). Figure 8 shows the echo time images from the irregularly spaced sequences, as well as signal magnitude for the marked ROI for the different subjects and sequences. Encouragingly, even this arbitrary echo spacing showed deviations from monotonic decay in the mildly steatotic liver (mean fat/water ratio of 18% measured using 3D Dixon VIBE), but not in the lean liver. In contrast, the evenly spaced echoes generated monotonic decay in both subjects. However, as predicted theoretically, the apparent rate of decay does change with echo spacing, even in the absence of fat. In lean liver, apparent T2 changes from 125 ms, for the uniformly spaced experiment, to 33 ms, for this particular nonuniform spacing. In steatotic liver, it changed from 151 ms to 41 ms. This further confirms the important role that J-coupling plays in apparent T2 decay, since a traditional decay curve would not be affected by echo spacing. Deviations from monotonic decay observed in nonuniformly spaced trains are also driven by J-coupling present in fat, and thus reflect the chemistry of those depots. Furthermore, it shows that unevenly spaced echo trains are a fundamentally different contrast mechanism than regular T2 decay—that is a J-coupling contrast.

## Discussion

These studies demonstrate with simulations, *in vitro* experiments, and even preliminary *in vivo* experiments, the basic phenomenon that irregularly spaced echo trains can generate complicated and distinctive echo patterns that reflect chemical composition. This result is expected from long established theory, but this work is the first to explore this as a means of chemical identification. An echo spacing optimized to distinguish particular lipids or diseases could potentially generate echo patterns that, via pattern matching analysis, could generate a disease score to aid diagnosis.

Given the global rise in obesity there is increasing recognition of the role of fat in health and disease^23,24^. One potential application is in the measurement of TAG vs DAG in fatty liver. Extensive work in mice and humans concludes that while TAGs are a relatively neutral form of lipid storage, DAG accumulation in liver activates protein kinase C isoforms, directly triggering hepatic insulin resistance, which eventually leads to more serious diseases such as Type 2 diabetes and liver disease^19–21^,^25–27^, Liver biopsy samples show a stepwise trend in DAG/TAG ratios between healthy liver, benign steatosis, and nonalcoholic steatohepatitis (NASH)^18,28^. Therefore, the characterization of fat chemistry via J-coupling contrast could be an important component of a multiparametric MRI exam to distinguish benign steatosis from NASH^29^.

One potential concern is whether fatty acid chains will complicate or overshadow the phenomenon demonstrated for glycerol backbones. Though it is subject to the same basic phenomenon, this component of the molecule has a far more complicated J-coupling structure that may be more difficult to tease apart through J-coupling dynamics. However, since the spins in the fatty acid chain primarily resonate below 4 parts per million (ppm), a crusher pulse suppressing that part of the spectrum could improve contrast based on glycerol backbones.

Another significant concern may be whether B0 or B1 variations will confound results. Most B0 variation does not change Δω between coupled spins and is not expected to affect spin echo signal intensity at all, as we have verified with simulations. Additional signal loss due to diffusion through B0 gradients should be comparable within a given disease state and thus would be built into the signal model, though an additional diffusion or T2 map could also be used as a correction factor. Simulations do suggest that a relatively small subset of potential echo spacings exhibit significant B1 sensitivity. For human studies, an additional scan may be needed to generate a B1 map so that flip angle can be tuned to high accuracy over the ROI targeted for analysis^30,31^.

In summary, this work introduces a new approach to the molecular characterization of J-coupled species, such as fat. The approach has the advantage of being insensitive to B0 and easily incorporated into an imaging sequence. Irregularly spaced echo trains could be used to generate a signal vector which can be decomposed into relevant basis functions, or they can also be used as a contrast preparation module to target the echo of greatest contrast. This approach to distinguishing fats could ultimately find a broad range of biomedical applications, such as distinguishing DAG and TAG in liver, imaging lipid based therapeutics, or detecting other lipotoxic subtypes.

## Methods

### Simulation Algorithm

Density matrix simulations were performed using version 1.6.2782 of Spinach using spin parameters shown in Table 1 at a field strength of 3 T^32,33^. Simulated experiments used a 16 echo train with echoes always acquired symmetrically about the 180. More specifically, each pulse sequence was defined by a 16 element array of delays (τ_i_). Following the initial 90° excitation pulse, each sequence consisted of 16 concatenated modules of the form: τ_i_ /2 − 180° RF − τ_i_ /2 – echo, with the acquisition window centered around the echo, thus starting slightly before τ_i_ /2. Each echo consisted of 128 points acquired over 1.3 ms to minimize the effect of chemical shift. To test off-resonance effects on B1, the broadband refocusing pulse was replaced with a 170° pulse on H3 of Mol B and a 180° pulse on all other protons. For each pulse sequence, the array of delays was generated by a random selection from a Gaussian distribution with the indicated mean and standard deviation, taking absolute values to avoid negative delays. Each pulse sequence was simulated on each molecule, and the magnitude of the two resulting 16 echo signals was analyzed for correlation. Low correlation between the signals indicates potential for good contrast.

**Table 1:** Spin parameters used for simulations with Mol A and Mol B.

	H1 (ppm)	H2 (ppm)	H3 (ppm)	H4 (ppm)	H5 (ppm)	R2 sec^−1^	R1 sec^−1^	J Couplings (ppm)
Mol A	4.13	4.18	4.23	4.13	4.18	30	1	J_13_ = 4.0 J_23_ = 3.0 J_34_ = 4.0 J_35_ = 4.5 J_51_ = 3.0
Mol B	4.29	4.29	5.26	4.29	4.29	10	1	J_13_ = 2.0 J_23_ = 4.0 J_34_ = 6.0 J_35_ = 6.0 J_51_ = 8.0

These are roughly modeled on the glycerol backbones of diacylglycerols and triacylglycerols, in that they are isolated 5-spin networks with very similar chemical shifts.

To test whether signals depended primarily on average echo spacing in an irregularly spaced sequence, density matrix simulations were performed for sets of ten sequences, all with 16 echoes whose spacings were randomly chosen from a single Gaussian distribution (same mean ESP, or μ(ESP), and standard deviation, with absolute values taken to avoid negative delays). Each sequence was simulated on two 5-spin molecules modeled based on the glycerol backbones of DAG and TAG, referred to as Mol A and Mol B, respectively. Contrast between the species was quantified as correlation between the two echo-train signals. The CMPG sequence was included as the case where StdDev(ESP) = 0. For every sequence that generated correlation <0.9, the sequence was simulated again assuming a global B0 shift of +/−0.5 pp, as well as with global T2 multipliers of 0.7 to 1.3 in increments of 0.1 and with B1 discrepancies of +/−10%.

### Phantom Studies

All MRI experiments were performed on a 3 T Siemens Trio magnet with a birdcage receiver coil. Bottles were prepared with arrays of diacetin/triacetin oil percentages of 100/0, 50/50, 10/90, 5/95 and 0/100 and arrayed along either the x or y axis of the magnet, so that bottles would not overlap in a 1D projection. Bottles were imaged with a spin echo train with 16 echoes, repeated with 64 steps of 1D phase encoding corresponding to a 20 cm field of view (FOV). Therefore, the entire timeline of each freely evolving echo (128 points acquired over 1.3 ms per echo) was acquired from each bottle. Repetition time (TR) was 2 s, and echo time (TE) spacing was either 16 ms across the echo train, or else followed a pattern of 25-6-6. Single voxel spectra were also collected from each bottle using the single voxel spectroscopy sequence after shimming on each volume individually. Other scan parameters for the spectroscopy experiment were TR/TE/Ns/BW/FOV = 2 s/30 ms/512/1 kHz/2 cm × 2 cm × 2 cm. The entire experiment was repeated on a different day with bottles placed in a different order and orientation. Signals from each bottle were then quantified by decomposition using the signals from pure diacetin and triacetin acquired in the first experiment.

### Human Studies

For human images, subjects were imaged on the same 3 T scanner with PACE navigator gating monitoring the diaphragm lung interface. Images were acquired with a Siemens spin echo train imaging sequence, modified to acquire 16 contrasts with nonuniform timing. Intensity in each image is expected to reflect magnitude of the echo peak at each echo time. All experimental protocols were approved by the Yale Institutional Review Board (Protocol #1109009027), and informed consent was obtained from all subjects, who were adults, as described in the protocol. All procedures in these studies followed the relevant guidelines and regulations. Scan parameters were TR/SliceThickness/FOV/Resolution/BW = 3000/5 mm/38cmx30cm/128 × 51/781, using echo spacing 25-6-6 or a uniform spacing of 16 ms.

## Data Availability

All data generated during the current study can be made available from the corresponding author upon request.
